# A novel three-dimensional printed vacuum bell for pectus excavatum treatment: a preliminary study

**DOI:** 10.1186/s13019-020-01276-y

**Published:** 2020-09-10

**Authors:** Xicheng Deng, Peng Huang, Jinwen Luo, Jinghua Wang, Liwen Yi, Guangxian Yang, Siping He, Xun Li, Shiting Xiang

**Affiliations:** 1 Deaprtment of Cardiothoracic Surgery, Hunan Children’s Hospital, No. 86 Ziyuan Road, Changsha, 410007 Hunan China; 2 Deaprtment of Radiology, Hunan Children’s Hospital, Changsha, 410007 Hunan China; 3 Pediatrics Research Institute of Hunan Province, Hunan Children’s Hospital, 86 Ziyuan Road, Changsha, China

**Keywords:** Pectus excavatum, Vacuum bell, 3D printed

## Abstract

**Objective:**

Conservative treatment with a vacuum bell (VB) for pectus excavatum (PE) has now been gradually popularized as an alternative to surgery. We describe our initial experience with a novel three dimensional (3D) printed VB device.

**Methods:**

Prospectively collected data of all patients who started using a 3D printed VB in 2018 at our institution were analyzed. Linear and logistic regressions were used to identify factors associated with effectiveness of device usage.

**Results:**

In total, forty-two patients with a median age of 3.6 years were treated with the device. The median follow-up duration was 11.1 months and the mean initial Depth Ratio (DR) was 0.129. There were no permanent sequelae from side effects. Thirty patients with at least one follow-up body scan data showed varying improvement (z = − 4.569, *p* = 0.0000). Linear regression suggested that longer usage improved outcomes (R^2^ = 0.235, *p* = 0.014). By logistic regression there was a trend of younger ages and less initial DR for better improvement though neither was statistically significant (*p* = 0.086, 0.078, respectively).

**Conclusion:**

Our initial experience has shown the 3D printed VB may be as effective as other conventional VBs and could be used as an alternative to surgical treatment for selected patients with PE. More experience and studies with this type of VB are needed to demonstrate its superiority with regard to the 3D printing design and optimal timing and indication for use.

## Background

The vacuum bell (VB) has established its role in the treatment of pectus excavatum (PE) in the last 20 years [1–6] as an alternative to surgery it has been extensively reported by Haecker, et al. [1–3]. And then its usefulness has been confirmed by other authors [4]. However, all their VBs are premade with fixed sizes and shape. In this study, we tried to incorporate the unique configuration of the chest wall deformity of each and every individual for the manufacture of a VB. We started to use a three dimensional (3D) printed VB bespoke for every patient since 2018. To the best of our knowledge, this is the first one of its kind incorporating 3D technique. Here we report our initial experience with this novel VB and short-term follow-up results.

## Methods

### The manufacture of a 3D printed VB

**Fig. 1:**
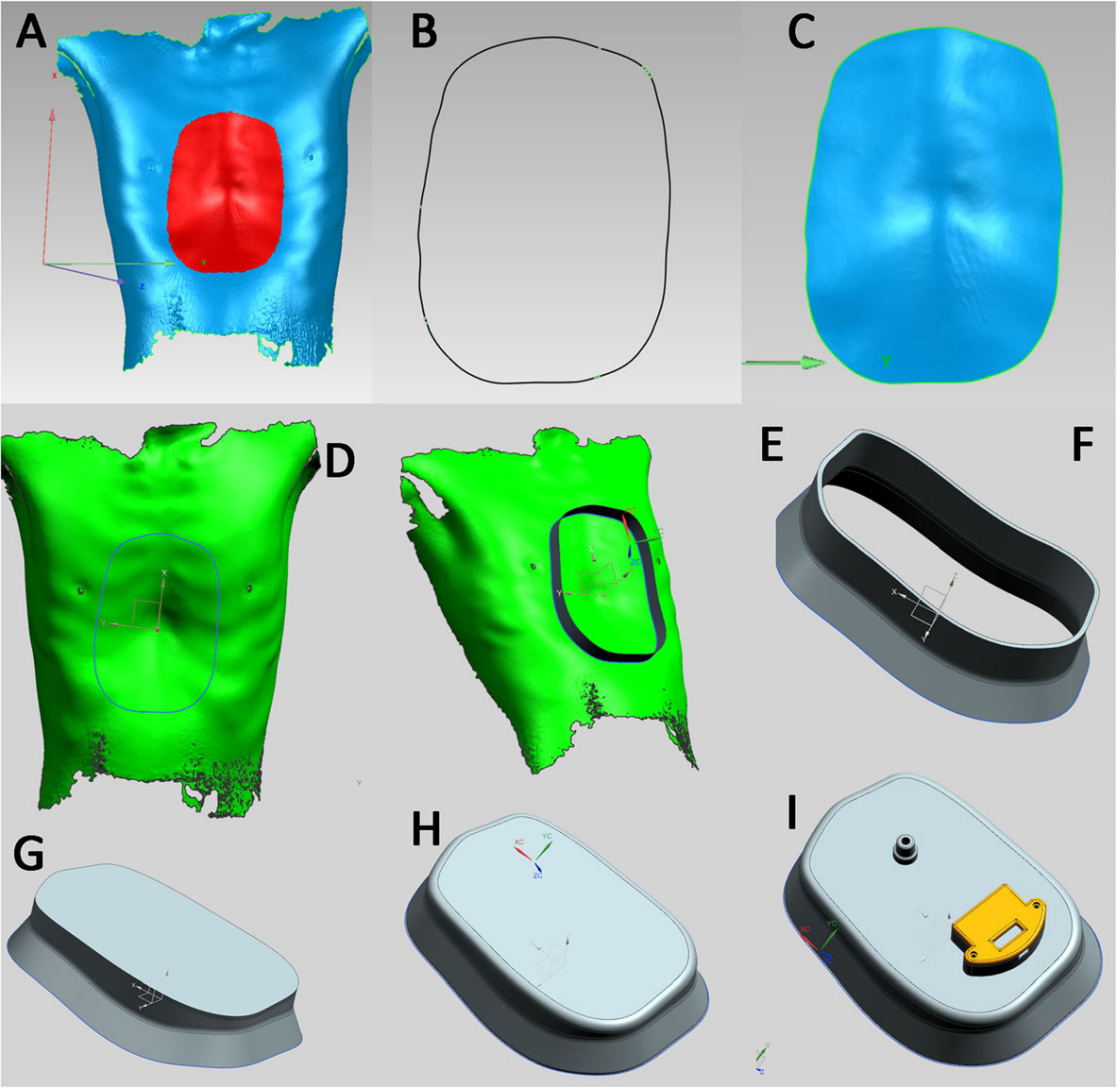
The steps to design and manufacture a customized silicon part of the 3D printed VB

**Fig. 2:**
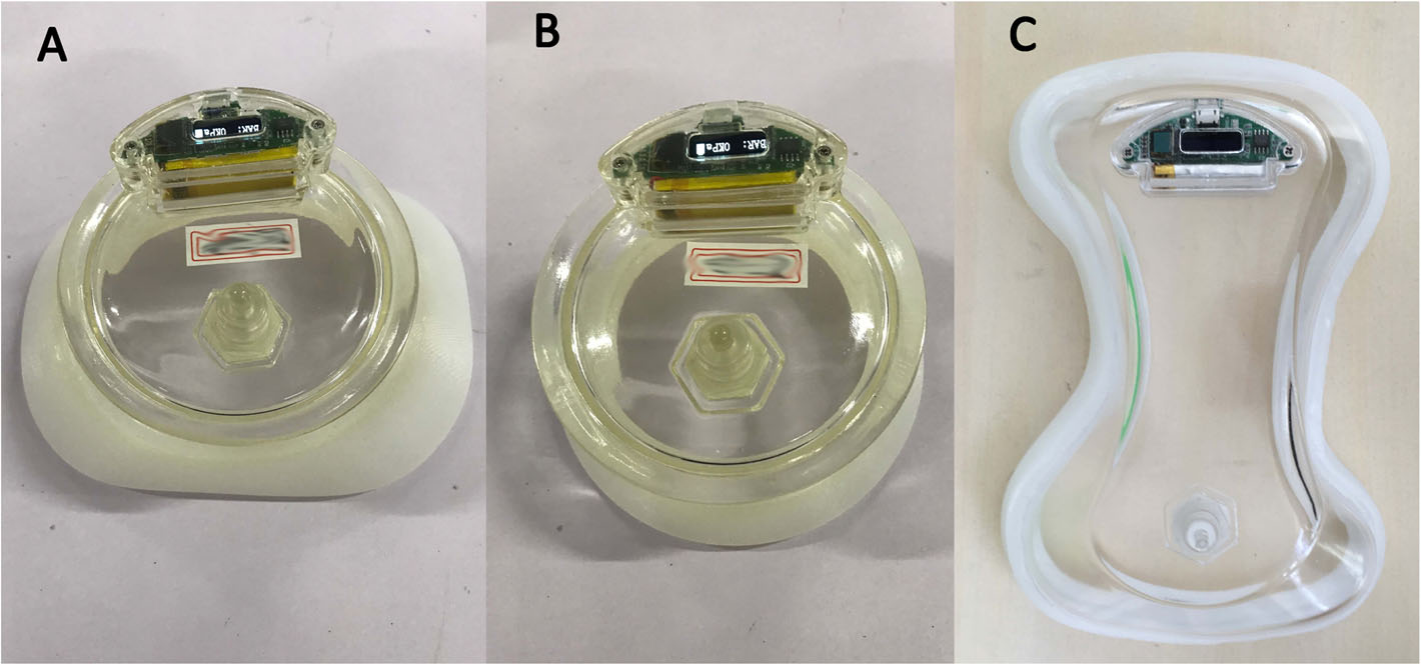
Different shapes of the 3D printed VBs

The 3D printed VB (Kenvoxel Co.,Ltd. Guangzhou, China) is manufactured involving the optical body surface scan steps and has been described in detail in our previous publication [7]. Once data has been obtained with body surface scan, the images are imported to Geomagic Studio (3D Systems®, Rock Hill, SC, USA) to extract the funnel area, ready for design of the silicon part (skin contact part) (Fig. 1). The silicon part is manufactured and connected to a top part with an electronic pressure gauge and a check valve (for hand pump connection) mounted and ready for use. (Fig. 2).

### The measurement of PE severity

**Fig. 3:**
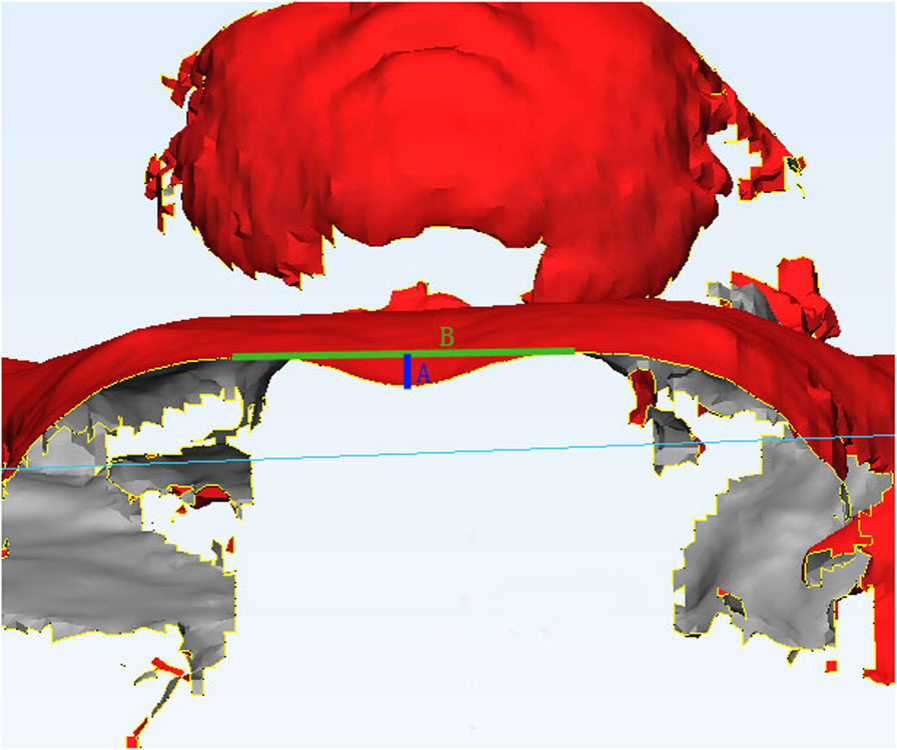
The measurement of the Depth Ratio, defined as a/b. **b**: the distance between the highest points on each side of the front chest; **a**: the shortest vertical distance between external deepest point of the front chest and b

Only part of the patients received chest computed tomography (CT) scans and the Haller Index were calculated. Besides, all the patients underwent front chest-only optical body surface scan in supine position, in which case the Haller Index could not be obtained and the Depth Ratio (DR) (Fig. 3) were measured as previously described [7] on body surface scan image instead. Every patient underwent initial scan upon ordering of a VB and later follow-ups every 3 months to half a year during use of the device.

### Study enrollment and usage of the VB

Approved by institutional review board and patient consent waived, a retrospective study evaluating outcomes of nonsurgical treatment PE with the 3D printed VB was performed. Patients were enrolled from March, 2018 to December, 2018. Indications for VB therapy were moderate to severe PE by inspection or CT scan yet too young for surgery, or recurrence after surgery, or aversion to surgery. Contraindications to VB therapy were coagulopathies. All potential candidates for VB treatment were seen and approved or disapproved by a single surgeon. After approval, patients received body surface scan. The collected body surface data were used for VB customization as well as deformity documentation in order to compare with follow-up scans during the course of treatment. By filing online questionnaires one and a half year after the initiation of the study, the patients reported the status of daily VB use (average duration in minutes and times daily), the duration of the treatment (accumulated months used), the vacuum pressures (kPa). The vacuum pressure ranges were recommended according to patients’ ages. The VB was first applied 15 min twice daily, and then increased to 30 min twice daily after 2 weeks or more. The whole course of treatment was 1 year and might be extended as appropriate.

### Statistical analysis

Patient demographics, the Haller indices and DRs were collected for statistical analysis. Continuous data following normal distribution were presented in mean ± standard deviation, or in median and interquartile range if not following normal distribution. Categoric data were present in frequencies and percentages. The initial DRs and last follow-up DRs were compared using Wilcoxon Signed-rank test or Student *t* test as appropriate to identify improvement. A broken line plot was also drawn to visualize change in DR over time for patients having had at least one follow-up scan. The correlation between usage duration of the VB and change in DR (last follow-up DR subtracted by initial DR) over the duration was visualized in scatter plot and analyzed using linear regression. Logistic regression was used to identify factors associated with better effects with VB use. The dependent variable Post-correction Ratio was defined as the ratio of the least DR measured during follow-ups to the initial DR. A cut-off of 0.5 was chosen to transform it into a dichotomous variable. The Post-correction Ratio less than 0.5 were considered more effective of VB use. In univariate analysis *P* < 0.1 was chosen as inclusion criteria for multivariate step and in multivariate analysis, *P* < 0.05 was considered significant. All analyses were conducted using Stata version 15 statistical software (Stata Statistical Software: College Station, TX: Stata Corp LP).

## Results

Forty-two patients (female 16,38.10%) with a median age of 3.6 (2.2,6.2) years received nonsurgical treatment with the device. The mean DR at treatment onset was 0.129 ± 0.036. Eighteen subjects had undergone a chest CT scan with a mean Haller index of 3.07 ± 0.42 (Table 1).

**Table 1:** Demographics of 42 patient receiving vacuum bell therapy

	Mean ± SD/median (IQR)/frequency, percentage
Age, years	3.6 (2.2,6.2)
Female	16,38.10%
Height, cm	106.3 ± 21.4
Weight, kg	21.0 ± 8.2
Initial DR	0.129 ± 0.036
Haller index	3.07 ± 0.42

Forty patients/parents completed the questionnaire upon request. The mean follow-up duration was 11.1 ± 3.8 months according to the last body scan date, and change in the DR was − 0.057 ± 0.023. With regard to complications, 21(52.5%) complained petechiae in use, and 12(30%) reported blistering and some reported pain and/or discomfort. All the symptoms resolved after short time pause. There were no permanent sequelae from side effects. With regards to effectiveness, the majority considered it good (20, 50%) or fair (18, 45%). Two patients/parents considered it excellent (Table 2). All patients were still using the VB if not asked to pause or terminate.

**Table 2:** Follow-up and questionnaire results

	Frequency/median (Percent/range)
Questionnaire responders	40 (100%)
How was the device used?	
Duration per time, minutes #	30 (15,60)
TIMES per day	1.5 (1,3)
Vacuum, kPa	20 (10,30)
Complications	
Petechia	21 (52.5%)
Blistering	12 (30%)
Discomfort	5 (12.5%)
Pain	9 (22.5%)
Effectiveness by questionnaire	
Poor	0 (0%)
Fair	18 (45%)
Good	20 (50%)
Excellent	2 (5%)
Follow-up	Mean (SD)
Duration, months	11.1 (3.8)
Change in DR	−0.057 (0.023)
Ratio^a^	0.54 (0.20)

# Duration may have changed during treatment. Patients or parents were required to report the parameters they used most

^a^ The ratio of the least DR during usage to initial DR

**Fig. 4:**
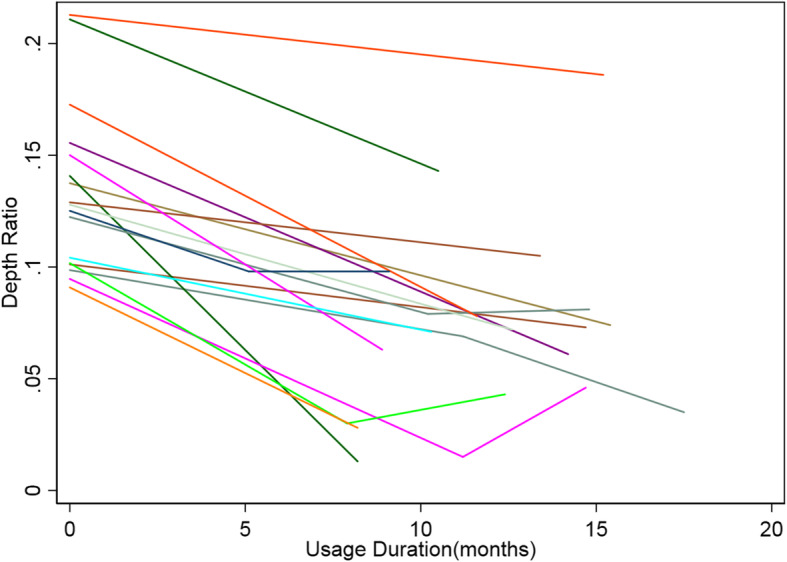
A broken line plot showing changes in DR over the course of treatment

**Fig. 5:**
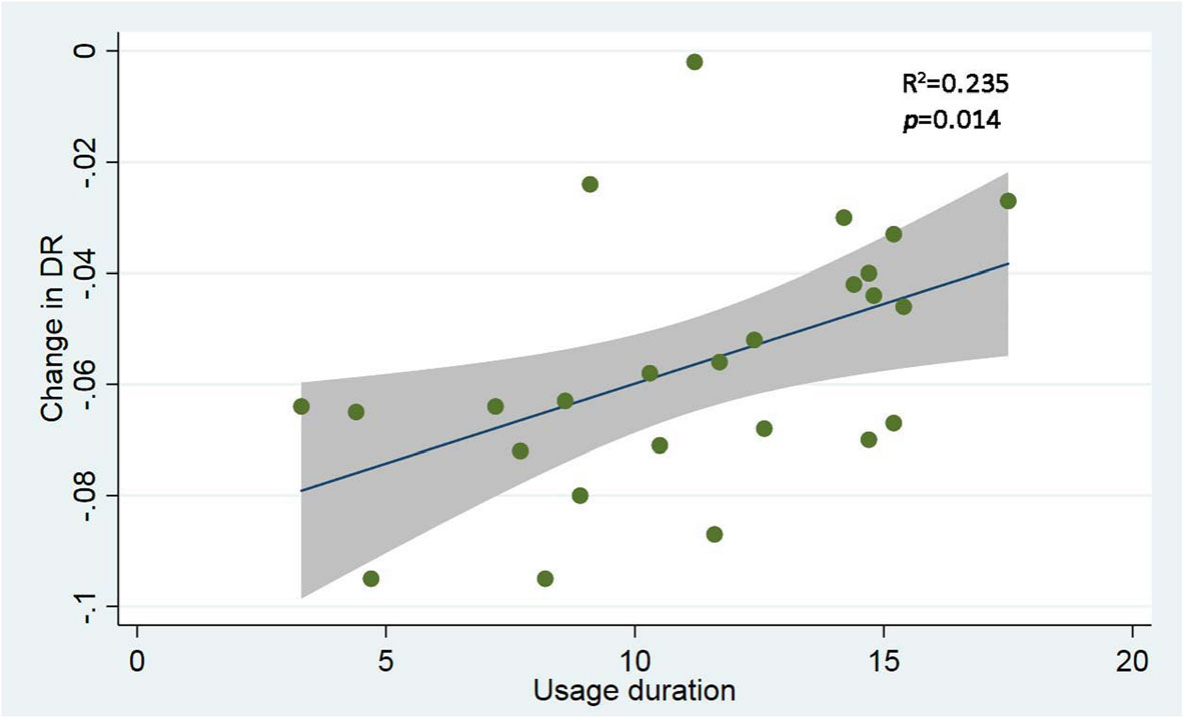
The scatter plot and fitted line shows a correlation between usage duration and change in DR, R^2^ = 0.235, *p* = 0.014 by linear regression

**Fig. 6:**
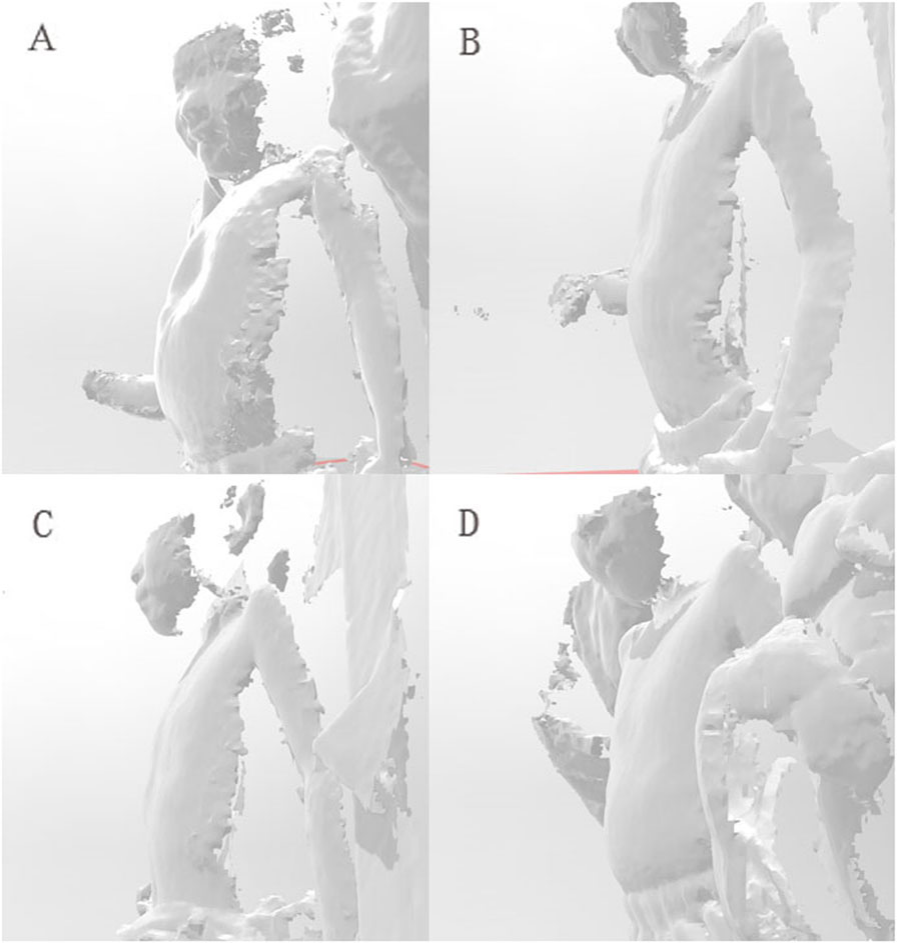
An example of typical patients using 3D printed vacuum and having demonstrated significant improvement of the depression of the front chest. **a**: Started to use the 3D printed VB; **b**: 3 months after use; **c**: 9 months after use and stopped VB therapy; **d**: 3 months after termination of VB use

There were 30 patients having had at least one follow-up scan and Wilcoxon Signed-rank test shows significant improvement after use of the VBs (z = − 4.569, *p* = 0.0000). All patients achieved better appearance after use though there was some fluctuation as show in the broken line plot (Fig. 4). The scatter plot and fitted line shows correlation between usage duration and change in DR (R2 = 0.235, *p* = 0.014 by linear regression, Fig. 5). Significant improvement of the front chest depression of an typical patient using 3D printed vacuum is demonstrated with reconstructed images using body surface scan technique (Fig. 6). After exclusion of 12 patients with no follow-up scan and 5 patients with 10 week or longer pauses, 25 patients with available follow-up scan data were included for logistic regression. In univariate analysis, p for age and pre-treatment DR was < 0.1 and were included in multivariate analysis. However, none of the two factors was significant in the next step analysis (*p* = 0.086, 0.078, respectively) (Table 3). At the conclusion of this study, 3 achieved normal front chest appearance and terminated the VB therapy. They were required to visit 3 months later after discontinuation. The rest were still using the device.

**Table 3:** Logistic regression for better corrective effects of PE

Variable	Univariate			multivariate		
	OR	95%CI	*p* value	OR	95%CI	*P* value
Age	1.37	(0.96,1.95)	0.084	1.40	(0.95,2.07)	0.086
Sex	1.60	(0.33,7.85)	0.562			
Height	1.03	(0.99,1.08)	0.115			
Weight	1.07	(0.95,1.20)	0.268			
Initial DR	3.43e+ 11	(0.02,7.69e+ 24)	0.090	3.40e+ 11	(0.05,2.37e+ 24)	0.078
Duration between treatment onset and last follow-up	1.20	(0.95,1.51)	0.130			
Total hours of VB usage	1.00	(1.00,1.01)	0.425			
Vacuum pressure	1.02	(0.85,1.2)	0.854			
Times per day	0.60	(0.14,2.55)	0.491			
Hours of VB usage daily	1.05	(0.96,1.14,)	0.263			
Total days VB used	1.00	(0.99,1.01)	0.894			

## Discussion

Our study has shown the customized 3D printed VB therapy offers nonoperative treatment of PE at least as effectively as conventional VB though it remained unclear if it results in better outcome at the expense of 3D printing customization. All patients with follow-up data presented better chest appearance confirmed both objectively and subjectively.

Using vacuum suction to lift the sternum was first described more than a century ago, and its usefulness has been confirmed in the treatment of PE over the last 20 years [1–6]. Obviously, one advantage brought by VB therapy compared to surgery is fewer associated risks and less discomfort. However, it remains an alternative rather than a substitute for the Nuss procedure which can achieve excellent results in the majority of patients with one procedure and minimal complications at this stage [5]. A major shortcoming of the VB therapy is that it requires remarkable dedication by the patient and family to keep constant use of the device over at least 1 year or even longer [3] during which many are not able to maintain this compliance. Obermeyer, et al. [8] reported nearly one third of their patients were either lost to follow-up or poorly compliant. Our patients’ compliance seemed higher as we required them to visit us every 3 months to half a year since VB onset. And we also kept patients in touch in Wechat, a Chinese social media platform. Regular visits with objective measurement for improvement using body surface scan and inter-parent communication on social media encouraged them to continue the treatment. In the course of treatment as long as at least 1 year, it is very important to have patients back and show them the improvement in the form of reconstructed body surface images and measured numbers. Hence, patients and families may gain confidence in this novel therapy and maintain high compliance.

### Effectiveness and side effects

An excellent correction with VB therapy has been reported from 13.5 to 37.5% [1, 4, 8, 9] though different definitions of excellence were used. Some use absolute depression depth, which does not account for the body size of patients and requires special tools for measurement, to quantify the correctness, while others use Haller Index, Correction Index or the modified percentage depth [10], all of which require a chest CT scan or special calipers to measure dimensions on body surface. In comparison, as mentioned before, only part of our patients received CT scan and we only body-scanned the front chest of all patients due to insufficient compliance of young patients and also to simplify measurement procedures. Front-chest-only images are enough to customize a VB but makes it impossible to calculate external Haller Index or any other alternative indices. So we measured the DR which accounts for the width of the chest as compared to absolute depression depth. It is radiation free, reproducible and needs no additional resource or tools. We have demonstrated its consistency with data measured from chest CT in our previous study [7]. According to the DR, all patients with follow-up data improved in varying degrees. However, we did not use any cut-off of the DR for the definition of excellence outcome. This makes it difficult to compare excellence rates between our series and those in the literature. As per questionnaire data, the majority of the parents considered the effectiveness fair to good. Since most of the patients were still in the course of treatment, the effectiveness was expected to continuously improve. Side effects were as common as reported in the literature, including petechiae, blistering, discomfort or pain [1, 8, 11]. Although common, all side effects were reversible and resolved by applying reduced suction pressure or pausing for a short period of time, generally no more than 2 weeks. There were no side effects leading to noncompliance.

### Outcome related factors

Previous studies have related several factors with effectiveness of VB use. Obermeyer, et al. [8] found factors predictive of an excellent result being: initial age ≤ 11 years old, initial chest wall depth ≤ 1.5 cm, good chest wall flexibility, and usage duration more than 12 months. They noticed that several patients used below 60 min per day and still achieved an excellent correction, which was in contrast to Lopez et al., who identified optimal use of 4 h or more per day [4]. However, both of them argued that chest wall flexibility is a significant indicator of success. Though we did not find any factor associated with better correction of the PE, there was a trend for patients with younger age and smaller DR (less severity) to have better outcomes according to our data. And patients in our study much younger than those in the two publications mentioned above may explain why we had achieved remarkable improvement with only 30 min per time and 1.5 times daily use, which was much shorter than anyone reported before. We did not included flexibility as an analytical factor as first, it was difficult to quantify it and second, we usually consider the chest of a population with a median age of 3.6 years flexible, which was evidenced by a median suction vacuum of only 20 kPa. Obviously, chest flexibility is closely associated with age. It could not be ruled out that these two may interact as confounders when performing regression analysis, especially when all previous studies are of small sample sizes.

### Timing of treatment

With regard to timing for treatment, it once was indicated for preschoolers to have Nuss procedure and then switched to teenagers/adolescents as an optimal age for surgery in view of higher recurrence if patients were operated on very young [12]. For VB therapy, Obermeyer, et al. [8] concluded VB therapy should be considered at approximately 11 years of age. Since if the correction is inadequate after 12 to 18 months of VB therapy, surgery could be performed in the optimal timing range for the Nuss procedure. We believe, however, a conservative therapy like VB can be tried as young as toddlers or preschoolers like our cases for current data support better outcomes in younger patients with more flexible chest, especially in patients with deep depression in whom treatment could not be refrained until later. Though we do not yet know the recurrence rate of our series due to short follow-up, it might be expected to have higher recurrence with experience from surgery at very young ages. However, a VB therapy is of minimum major complications, and could be repeated if necessary, without worry about osteochondrodystrophy or other major complications of surgery. It may be worth it to try at a young age to avoid years of waiting during which heart and respiratory impair may occur.

### Advantage of 3D and the cost

The prominent difference of the device we used in this study from conventional VBs, as we mentioned before, is the 3D printing customization. Basically, our device draws on the same physical principle as other VBs reported in the literature. Theoretically, a 3D contact surface configuration may warrant better airtightness in application, which is especially true in patients with breasts developing or asymmetrical pathology. We did not use a conventional VB for comparison in our study, and the configuration of the depression may have changed over the course of treatment, leaving the initial airtightness in question. Therefore, the superiority in airtightness of this devices needs to be confirmed in further studies. The cost of a customized VB is 6000 RMBs, approximately double the price of a conventional VB in China, but still much cheaper than a Nuss procedure, which would cost 25,000 RMBs. We think it is worth trying in view of published results of conventional VBs and the cost.

### Limitation

This study is limited in that it is a retrospective case series without control. And there were some patients who were lost to follow-up, poorly compliant, or had insufficient data. There were also some other limitations. First, questionnaire data with regard to daily use and pressure were self-reported and could not rule out memory-related or other errors. Second, the pressure applied varied according to age and stage of use, so relevant data were represented by values used most for each patient. Third, since our patients were generally young and the majority had passed through a winter during the course of treatment, quite a few paused for several weeks or even months. Though parents were required to report these pauses, errors might have occurred when calculating accumulated duration. Finally, as only part of the patients underwent chest CT and had the Haller index data available, we did not use the Haller index or depth for analysis. Instead, we used the DR as a surrogate, which has never been used in any of previous studies, making it impossible to conduct direct comparison with data in the literature.

## Conclusion

Our initial experience has shown the 3D printed VB may be as effective as other conventional VBs and could be used as an alternative to surgical treatment for selected patients with PE. More experience and studies with this type of VB are needed to demonstrate its superiority with regard to the 3D printing design and optimal timing and indication for use.

## Data Availability

The datasets used are available from the corresponding author on reasonable request.

## Abbreviations

PE: Pectus excavatum
VB: Vacuum bell
CT: Computed tomography
DR: Depth Ratio
3D: Three dimensional
